# Isobavachalcone Alleviates Plant Photosynthesis Inhibition Caused by Tobacco Mosaic Virus (TMV) Infection in Tobacco

**DOI:** 10.3390/plants14233638

**Published:** 2025-11-28

**Authors:** Lijie Guan, Yidan Wu, Wenli Sun, Mohamad Hesam Shahrajabian, Yuan Gao

**Affiliations:** 1 College of Environmental and Safety Engineering, Shenyang University of Chemical Technology, Shenyang 110142, China; 15640200918@163.com; 2 National Key Laboratory of Agricultural Microbiology, Biotechnology Research Institute, Chinese Academy of Agricultural Sciences, Beijing 100086, China; sunwenli@caas.cn (W.S.); hesamshahrajabian@gmail.com (M.H.S.); 3 School of Life Sciences, Jilin University, Changchun 130012, China; gaoyuan4796@163.com

**Keywords:** isobavachalcone, photosynthesis characteristics, proteomics, tobacco, tobacco mosaic virus

## Abstract

Viral infection affects photosynthesis in plants, significantly reducing crop yield and quality. This study investigated the effects of isobavachalcone (IBC), a natural compound extracted from the plant *Psoralea corylifolia* L., on photosynthesis in tobacco under tobacco mosaic virus (TMV; species *Tobamovirus tabaci,* family *Virgaviridae*) stress. TMV infection significantly reduced the chlorophyll content, Rubisco activity, net photosynthetic rate (Pn), stomatal conductance (Gs), intercellular CO_2_ concentration (Ci), and transpiration rate (Tr) in tobacco leaves. However, exogenous application of IBC (40 mg/L) effectively alleviated the negative impacts of TMV. Compared with the inoculated control, IBC treatment increased the chlorophyll content by 16.00–100.68%, enhanced Rubisco activity by 3.72–115.84%, and improved Pn, Gs, Ci, and Tr by 155.65–347.65%, 89.43–408.00%, 17.51–56.18%, and 106.76–336.80%, respectively, at 3–9 days post inoculation (dpi). Tandem massed tag-based quantitative proteomics analysis revealed that IBC upregulated the abundance of photosynthesis-related proteins, including those involved in photosystem II, cytochrome b6/f complex, photosystem I, and ATP synthase, under TMV infection. GO and KEGG enrichment analyses further confirmed that IBC enhanced the expression of proteins associated with photosynthesis and energy production pathways. These findings suggest that IBC can mitigate TMV-induced photosynthesis inhibition by increasing photosynthetic pigment content, promoting carbon assimilation, and regulating photosynthesis-related proteins, providing new insights into the molecular mechanism of IBC-mediated plant protection.

## 1. Introduction

Photosynthesis is a primary target of viral infection in plants. Viruses can interfere with plant photosynthesis through various mechanisms. First, viruses can induce chlorophyll degradation, leading to chloroplast damage and reducing chlorophyll content, thereby affecting photosynthesis [1,2,3]. Liu et al. [4] demonstrated that the contents of chlorophyll a and chlorophyll b in virus-infected cowpea decreased by 32% and 40%, respectively [4]. Maravi et al. [5] found that plants infected with mungbean yellow mosaic India virus (MYMIV; species *Begomovirus vignaradiataindiaense*, family *Geminiviridae*) exhibited a significant reduction of 27–55% in chlorophyll a, b and total chlorophyll content. Abdelkhalek et al. [6] discovered that the total chlorophyll content in faba beans infected by alfalfa mosaic virus (AMV; species *Alfamovirus AMV*, family *Bromoviridae*) decreased by 1.2%. Zheng et al. [7] demonstrated that TMV infection in tobacco leads to structural damage in chloroplasts, reduced chlorophyll synthesis, and abnormal leaf development, accompanied by chlorosis and yellowing. Chlorophyll content was significantly reduced in the leaves of tobacco plants infected with TMV.

Second, viral infection can also affect proteins that play crucial roles in plant photosynthesis, such as light-harvesting complex (LHC) proteins responsible for absorbing and capturing light energy [8]. Studies have revealed that the symptoms of leaf yellowing, curling, and delayed growth and development in plants infected by tomato yellow leaf curl virus (TYLCV; species *Begomovirus coheni*, family *Geminiviridae*) are caused by significant downregulation of genes encoding LHC proteins [9]. The content of LHC proteins in leaves infected by pea seed-borne mosaic virus (PSbMV; species *Potyvirus pisumsemenportati*, family *Potyviridae*) is significantly reduced [10]. Wang et al. [11] found that in poplar infected by bean common mosaic virus (BCMV; species *Potyvirus phaseovulgaris*, family *Potyviridae*), the expression of differentially expressed genes (DEGs) related to photosynthetic antenna proteins and photosynthesis pathways was completely or almost completely suppressed, indicating that BCMV infection reduced the photosynthesis of the host.

Furthermore, the four membrane protein complexes responsible for driving electron transfer in photosynthesis, including Photosystem II (PSII) complex, cytochrome b6/f complex, Photosystem I (PSI) complex, and ATP synthase (ATPase), are also affected during viral infection [12,13,14,15]. Hao et al. [16] demonstrated that in maize infected by maize chlorotic mottle virus (MCMV; species *Machlomovirus zeae*, family *Tombusviridae*) and sugarcane mosaic virus (SCMV; species *Potyvirus sacchari*, family *Potyviridae*), 6 differentially expressed isoforms (DEIs) in the cytochrome b6/f complex, 16 DEIs in F-type ATPase, 26 DEIs in PSI, 30 DEIs in PSII, and 17 DEIs in the photosynthetic electron transport pathway were significantly downregulated. Studies have shown that viruses can affect the photochemical activity of plants by reducing the number of proteins involved in electron transport between PSI and PSII [17]. Viruses can also cause a simultaneous decrease in the accumulation and abundance of D1 and D2 proteins in the PSII core of plants, leading to photoinhibition, which in turn leads to a decrease in plant carboxylation activity and a reduction in the available reducing power for photorespiration [18,19,20]. The cytochrome b6/f complex catalyzes the rate-limiting step in thylakoid electron transport, and it is a key point in the regulation of photosynthesis. Viral infection interferes with the function of the cytochrome b6/f complex in plant cells. Transcriptome analysis revealed that in plants infected by citrus yellow vein clearing virus (CYVCV; species *Potexvirus citriflavivenae*, family *Alphaflexiviridae*), the expression of genes encoding cytochrome b6/f complex proteins was significantly downregulated, resulting in a slower electron transport rate in the light reaction and weakened photosynthesis [21]. ATPase is also inevitably damaged during viral infection. As the final component of the photosynthetic chain, ATPase catalyzes the synthesis of ATP [22]. It was found that the content of ATPase in tobacco leaves infected by potato virus Y (PVY; species *Potyvirus yituberosi*, family *Potyviridae*) was significantly reduced [23].

In addition, viruses also have a certain impact on the dark reaction stage of photosynthesis. Transcriptome studies have shown that viral infection can affect the expression of ribulose-1,5-bisphosphate carboxylase/oxygenase (Rubisco), a key enzyme involved in the Calvin cycle, hindering the normal progression of the entire Calvin cycle [24,25].

Photosynthesis is the foundation of plant growth and development, providing organic compounds and energy for plants. Its impairment by viral infection can severely affect plant yield and quality, making the mitigation of the impact of viruses on photosynthesis a challenging issue. Although viral infection has severe impacts on plant photosynthesis, some agents have been found to mitigate such effects [26]. Sofy et al. [27] demonstrated that, compared to inoculated controls, the application of melatonin (MLT) and salicylic acid (SA) increased the chlorophyll and carotenoid content in the leaves of infected plants. Under viral infection, MLT can also enhance the activity of Calvin cycle enzymes and improve photosynthetic carbon assimilation [28], while SA treatment accelerates the rate of plant photosynthesis [29]. Lucas et al. [30] indicated that melatonin can neutralize the adverse effects of pathogens on net photosynthesis rates, restoring them to levels comparable to those observed in healthy plants upon pathogen exposure. However, apart from these findings, agents capable of maintaining or even promoting plant photosynthesis under viral stress are currently uncommon.

Isobavachalcone (IBC) is one of the main components extracted from the seeds of *Psoralea corylifolia* L. IBC is a flavonoid compound biosynthesized via the phenylpropanoid pathway. This pathway is intrinsically associated with plant disease resistance, and IBC represents a key disease-resistant product derived from it. Notably, IBC not only exhibits direct antimicrobial activity but also elicits systemic resistance in plants. In agricultural production, IBC is commonly used for the prevention and control of plant diseases such as rice blast fungus, apple rot fungus, and cucumber bacterial angular leaf spot [31]. Studies have shown that IBC can regulate the activities of cell wall chitinase, glucanase, and glucan synthase in the rice blast fungus, disrupting the structure and function of the cell wall and cell membrane, and accelerating the death of the fungus [32]. Liu et al. [33] demonstrated that IBC at a concentration of 40 mg/L provided 75% control efficacy against TMV, while significantly stimulating the activities of defense enzymes including superoxide dismutase (SOD), peroxidase (POD), polyphenol oxidase (PPO) and phenylalanine ammonia-lyase (PAL) to induce systemic resistance. Zhang et al. [34] demonstrated that *Psoralea corylifolia* L. seed extract, with IBC as its primary active component, significantly promotes tobacco growth at a 2000-fold dilution. This treatment led to marked increases in plant height and aboveground and underground biomass, while concurrently enhancing both biomass accumulation and the activities of key physiological indicators including chlorophyll content, SOD, POD, and catalase (CAT) antioxidant enzymes. However, the molecular mechanism underlying the growth-promoting effect of IBC remains unclear, especially under the stress of viral infection. This study explores the mechanism of IBC’s growth-promoting effect on tobacco under TMV stress at the physiological, biochemical, and proteomic levels.

## 2. Results

### 2.1. Effects of IBC on Physiological and Biochemical Changes in Tobacco Leaves Under TMV Stress

**Figure 1:**
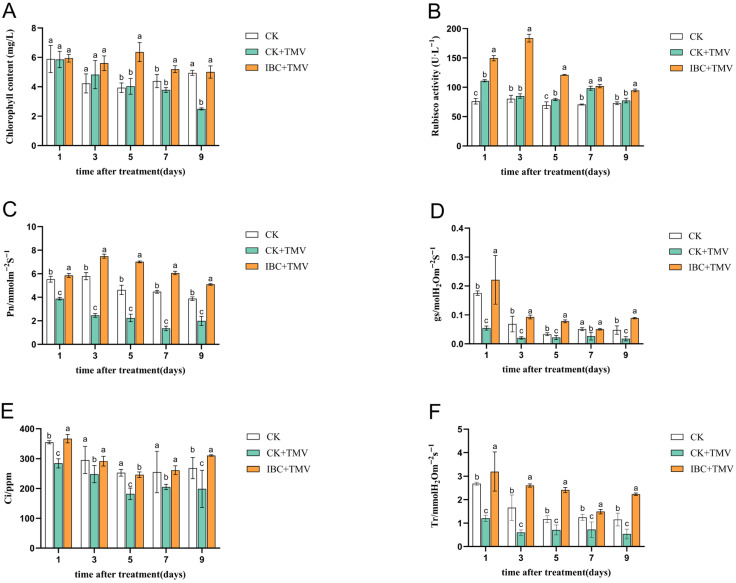
Physiological and biochemical responses in tobacco leaves after IBC + TMV treatment at various time points: (**A**) Chlorophyll content; (**B**) Rubisco activity; (**C**) net photosynthetic rate (Pn); (**D**) stomatal conductance (Gs); (**E**) intercellular CO_2_ concentration (Ci); (**F**) transpiration rate (Tr). Data are presented as the mean ± SD (*n* = 3 biological replicates). A two-way ANOVA followed by Tukey’s multiple comparisons test was performed to determine the effects of treatment and time. Different lowercase letters above the bars indicate significant differences (*p* < 0.05) among treatment groups at each time point.

As shown in Table 1, which details the symptomatic progression of TMV in NC89 tobacco, plants treated with IBC exhibited a significant delay in the onset of all symptoms compared to the CK + TMV. The experimental results (Figure 1) indicated that TMV inoculation caused varying degrees of decrease in chlorophyll content, net photosynthetic rate (Pn), stomatal conductance (Gs), intercellular CO_2_ concentration (Ci), and transpiration rate (Tr) in tobacco. Further comparative analysis of the experimental data among the CK group, CK + TMV group, and IBC + TMV group revealed that IBC treatment effectively alleviated the negative impact of TMV infection on the physiological indicators. Compared with the CK + TMV group, the IBC + TMV group showed a 115.8% and 336.8% increase in Rubisco activity and Tr on the 3rd dpi, respectively, a 347.7% increase in Pn on the 7th dpi, and a 100.7%, 56.2%, and 408.0% increase in chlorophyll content, Ci, and Gs on the 9th dpi, respectively.

### 2.2. Differential Expression Protein Analysis

**Figure 2:**
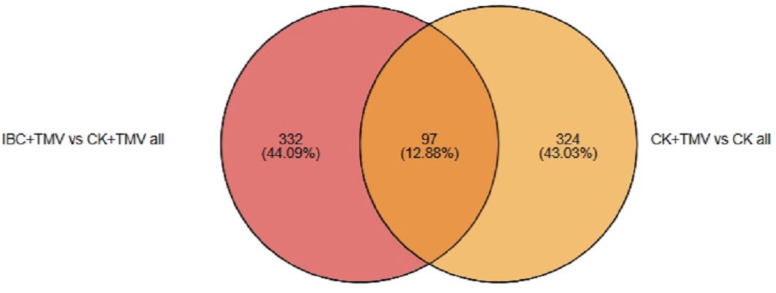
Venn analysis between protein sets.

**Figure 3:**
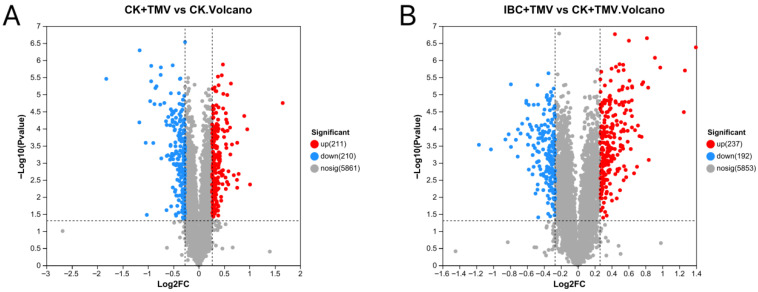
Volcano map analysis of significantly differentially expressed proteins (DEPs). (**A**) DEPs between CK + TMV and CK; (**B**) DEPs between IBC + TMV and CK + TMV.

Proteomic profiling was conducted at 24 h post-inoculation; the proteomic sequencing results showed that a total of 14,657 expressed proteins were detected in this analysis, of which 6282 proteins had quantitative information for further screening. DEPs between samples were analyzed based on the criteria of upregulated proteins with FC > 1.2, downregulated proteins with FC < 0.83, and *p* < 0.05. The Venn analysis of DEPs between different groups is shown in Figure 2. There were 421 DEPs between the CK and CK + TMV groups, including 211 upregulated proteins and 210 downregulated proteins. There were 429 DEPs between the IBC + TMV and CK + TMV groups, including 237 upregulated proteins and 192 downregulated proteins (Table 2). The number of common DEPs between the CK + TMV vs. CK and the IBC + TMV vs. CK + TMV comparisons was 97, while the number of unique DEPs was 324 and 332, respectively. The distribution of DEPs is shown in the volcano plot in Figure 3. The points farther from the center on the horizontal axis indicate a greater fold change, and the higher points on the vertical axis indicate a more significant difference. Subsequent bioinformatics analysis has been performed on all identified DEPs, including both up- and down-regulated proteins.

### 2.3. GO and KEGG Enrichment Analysis

**Figure 4:**
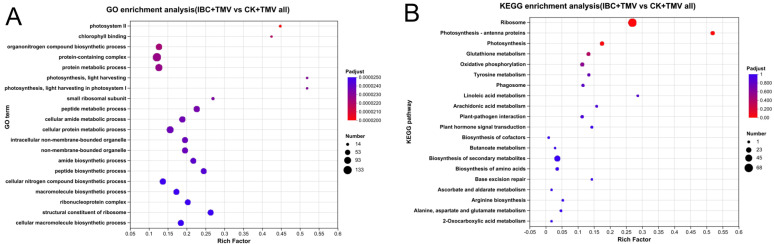
Enrichment analysis of DEPs in IBC + TMV vs. CK + TMV. (**A**) GO enrichment analysis of DEPs in IBC + TMV vs. CK + TMV. (**B**) KEGG enrichment analysis of DEPs in IBC + TMV vs. CK + TMV.

The GO enrichment analysis (*p*-adjust < 0.05) of DEPs between the IBC + TMV and CK + TMV groups showed that a total of 408 DEPs were annotated to 171 GO terms under three main categories: BP, CC, and MF (Table S1). The top 20 significantly enriched GO terms for DEPs are shown in Figure 4A, including the organonitrogen compound biosynthetic process (GO:1901566), protein metabolic process (GO:0019538), photosynthesis, light harvesting in photosystem I (GO:0009768), photosynthesis, light harvesting (GO:0009765), peptide metabolic process (GO:0006518), cellular amide metabolic process (GO:0043603), cellular protein metabolic process (GO:0044267), amide biosynthetic process (GO:0043604), peptide biosynthetic process (GO:0043043), cellular nitrogen compound biosynthetic process (GO:0044271), macromolecule biosynthetic process (GO:0009059), and cellular macromolecule biosynthetic process (GO:0034645) in the biological process category; photosystem II (GO:0009523), protein-containing complex (GO:0032991), small ribosomal subunit (GO:0015935), non-membrane-bounded organelle (GO:0043228), intracellular non-membrane-bounded organelle (GO:0043232), and ribonucleoprotein complex (GO:1990904) in the cellular component category; and chlorophyll binding (GO:0016168) and structural constituent of ribosome (GO:0003735) in the molecular function category.

The KEGG enrichment results showed that a total of 278 DEPs were enriched in 70 KEGG pathways (Table S2). The top 20 KEGG pathways are shown in Figure 4B. According to the criterion of *p*-adjust < 0.05, a total of 97 DEPs were significantly enriched in three KEGG pathways, namely the ribosome (nta03010, 68 DEPs), photosynthetic antenna proteins (nta00196, 14 DEPs), and photosynthesis (nta00195, 15 DEPs). These results suggest that under TMV stress, IBC mainly exerts its effects by regulating ribosomes, photosynthesis, and photosynthetic antenna proteins, among which photosynthesis and photosynthetic antenna proteins are closely related to plant growth.

### 2.4. Analysis of DEPs in the Photosynthetic Antenna Proteins Pathway

**Figure 5:**
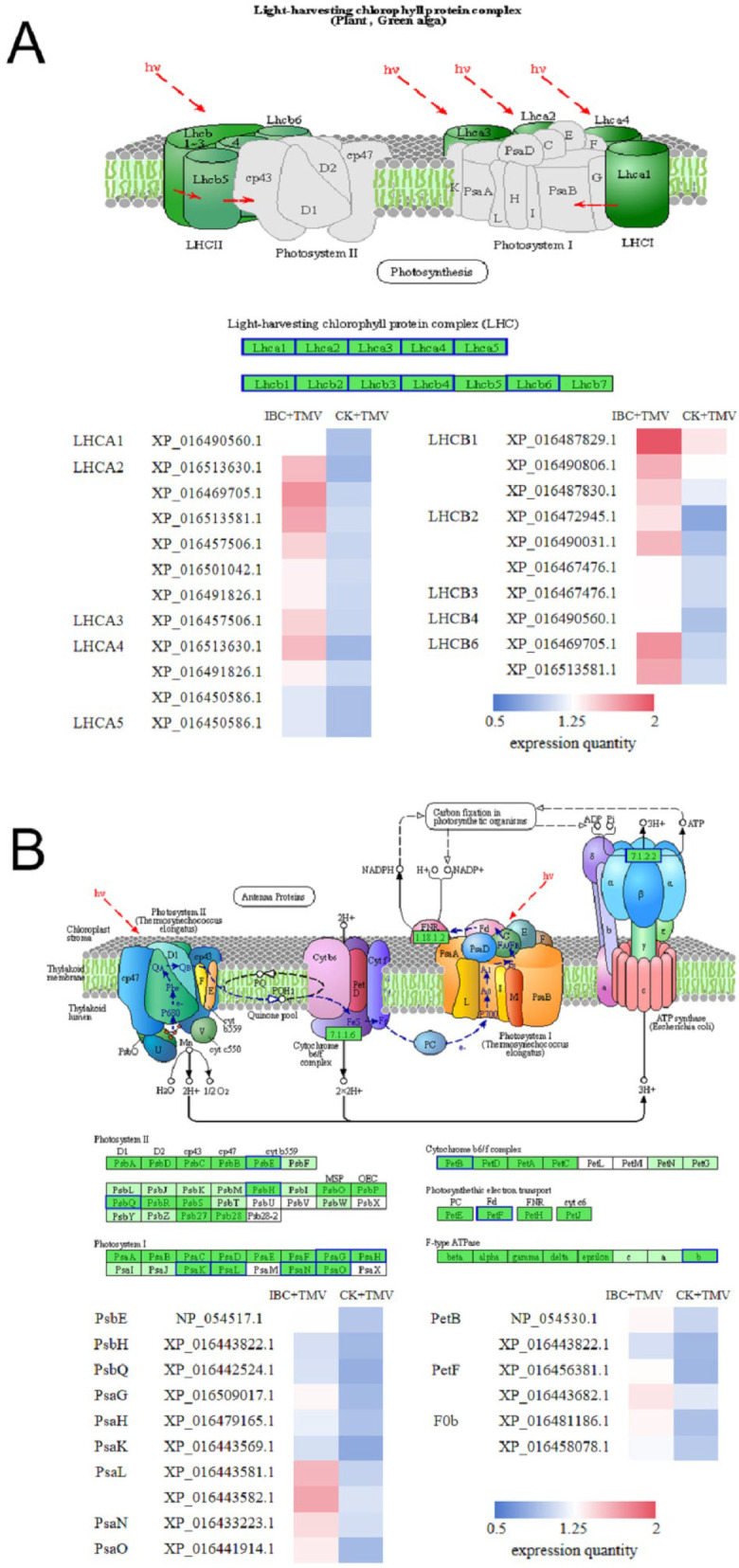
KEGG enrichment in photosynthesis pathways. (**A**) KEGG enrichment results in the photosynthetic antenna proteins pathway (nat00196). (**B**) KEGG enrichment results in the photosynthesis pathway (nat00195). A blue border in the pathway diagram indicates significantly upregulated expression levels.

**Figure 6:**
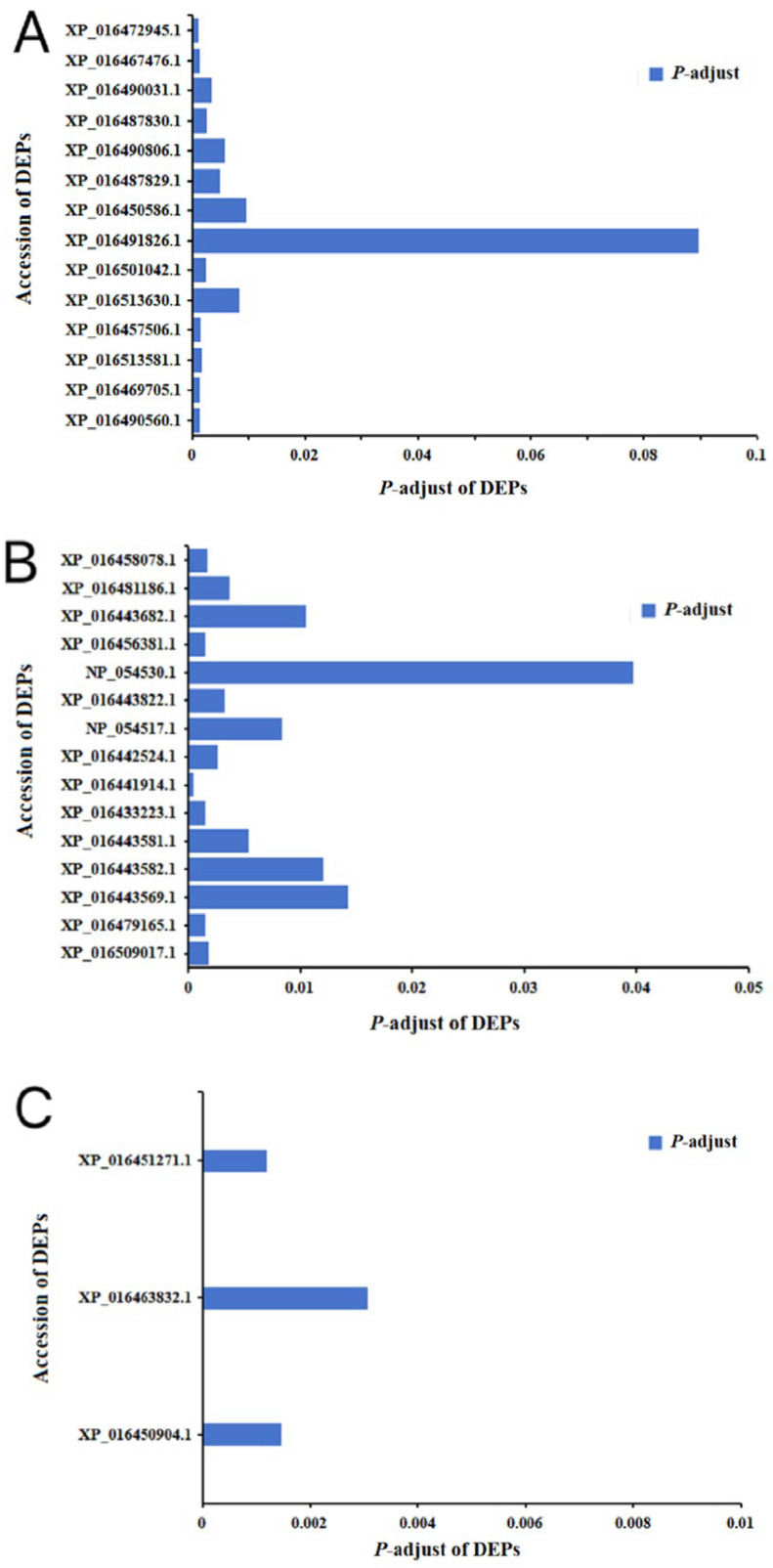
*p*-adjust of DEPs in photosynthesis-related pathways. (**A**) *p*-adjust of DEPs in photosynthetic antenna proteins. (**B**) *p*-adjust of DEPs in the photosynthesis pathway. (**C**) *p*-adjustment of DEPs in the photosynthetic pathway carbon fixation.

Photosynthetic antenna proteins play a role in absorbing and transferring light energy in plant photosynthesis, during the conversion of light energy to electrical energy, which is of great significance to photosynthesis. As shown in Figure 5A, there were 14 DEPs in this pathway between the IBC + TMV and CK + TMV groups. Eight DEPs were involved in the composition of plant PSI complex antenna proteins LHCA1 (XP_016490560.1), LHCA2 (XP_016469705.1, XP_016513581.1, XP_016457506.1, XP_016513630.1, XP_016501042.1, XP_016491826.1), LHCA3 (XP_016457506.1), LHCA4 (XP_016513630.1, XP_016491826.1, XP_016450586.1), and LHCA5 (XP_016450586.1). Nine DEPs were involved in the composition of plant PSII complex antenna proteins LHCB1 (XP_016487829.1, XP_016490806.1, XP_016487830.1), LHCB2 (XP_016490031.1, XP_016467476.1, XP_016472945.1), LHCB3 (XP_016467476.1), LHCB4 (XP_016490560.1), and LHCB6 (XP_016469705.1, XP_016513581.1). Simultaneously, differential expression analysis revealed that the expression levels of 14 DEPs in this pathway were significantly upregulated (Figure 6A and Table S3).

### 2.5. Analysis of DEPs in the Photosynthesis Pathway

Plants complete the conversion of electrical energy to chemical energy through electron transfer in photosynthesis, producing NADPH and ATP required for plant growth and development. In this pathway, there were 15 DEPs between the IBC + TMV and CK + TMV groups, all of which were involved in the PSI complex, PSII complex, cytochrome b6/f complex, and ATPase complex. As shown in Figure 5B, in this study, there were seven DEPs in the PSI complex, which were involved in the composition of six subunits: PsaG (XP_016509017.1), PsaH (XP_016479165.1), PsaK (XP_016443569.1), PsaL (XP_016443582.1, XP_016443581.1), PsaN (XP_016433223.1), and PsaO (XP_016441914.1), and their expression levels were all significantly upregulated. In the PSII complex, there were three DEPs involved in the composition of three subunits: PsbQ (XP_016442524.1), PsbE (NP_054517.1), and PsbH (XP_016443822.1), and their expression levels were all significantly upregulated. The DEPs involved in the composition of the PsbH subunit were also involved in the composition of the PetB subunit of the cytochrome b6/f complex. In the cytochrome b6/f complex, there were two DEPs (NP_054530.1, XP_016443822.1) involved in the composition of the PetB subunit, and their expression levels were significantly upregulated. In addition to the electron carriers mentioned above, there are four other proteins that play a role in electron transfer in plant photosynthesis, including plastocyanin (PC), ferredoxin (Fd), ferredoxin-NADP+ reductase (FNR), and cytochrome C6 (cytc6). In this study, there were two DEPs (XP_016456381.1, XP_016443682.1) involved in the composition of the ferredoxin petF subunit, and their expression levels were significantly upregulated. Furthermore, in ATP synthase, there were two DEPs (XP_016481186.1, XP_016458078.1) involved in the composition of the F0b subunit, and their expression levels were significantly upregulated (Figure 6B and Table S4).

### 2.6. Analysis of DEPs in the Photosynthetic Pathway Carbon Fixation

Green plants convert the chemical energy produced in photosynthesis into stable organic matter through carbon fixation. In this pathway, there were three DEPs between the IBC + TMV and CK + TMV groups, namely 5-phosphoribose isomerase 3 (XP_016451271.1), 5-phosphoribose isomerase 2 (XP_016463832.1), and Rubisco small subunit (XP_016450904.1), and their expression levels were all significantly upregulated (Figure 6C and Table S5).

## 3. Discussion

Photosynthesis plays a crucial role in the normal growth, development, and defense systems of plants. However, photosynthesis is affected by various abiotic and biotic stresses. Therefore, research on agents or compounds that can maintain or even promote plant photosynthesis under stress conditions has gradually become a hot topic. Studies have shown that IBC has the ability to promote alfalfa growth and induce antiviral resistance in tobacco [33,34], but the mechanism of its growth-promoting effects on plants remains unclear. In this study, we investigated the effects of IBC on tobacco photosynthesis under TMV stress by examining chlorophyll content, Rubisco activity, and photosynthetic parameters. Additionally, we explored the influence of IBC on photosynthesis-related proteins in tobacco under TMV stress using TMT-based quantitative proteomics technology.

Generally, the severe morphological and physiological changes observed in plants during viral infection are associated with alterations in chlorophyll content. Therefore, changes in chlorophyll content during viral infection can be used as an indicator of the functional status of plant photosynthesis [35,36]. Studies have shown that cucumber mosaic cucumovirus (CMV) infection reduces the chlorophyll content in cucumber plants [37,38,39], and TMV infection decreases the chlorophyll content in tobacco [40]. The reduction in chlorophyll content in virus-infected plants may be due to the virus downregulating chlorophyll biosynthesis (Chlorophyll synthase—*CHLG*) and upregulating chlorophyll degradation (Chlorophyllase—*CLH1* and *CLH2* and Pheophytin pheophorbide hydrolase—*PPH*) genes [41] or due to the virus affecting pigment synthesis, mineral absorption, and transport in plants [42].

Photosynthetic parameters are considered as a powerful tool for studying the physiological responses of plants to abiotic stress and are a direct method for assessing photosynthetic activity [43]. The main photosynthetic parameters include Pn, Gs, Ci, and Tr. Studies found that the Pn of host plants decreased under pepper mild mottle virus (PMMoV) infection also observed similar situations during bacterial infections [44,45,46]. Studies have shown that various viruses can cause stomatal closure and a decrease in Gs in plants [47,48]. In wheat infected with wheat streak mosaic virus (WSMV), Ci was significantly reduced. Furthermore, viral infection indirectly led to a decrease in plant Tr, weakening the plant’s cooling ability and subsequently amplifying plant heat stress during viral infection [49]. The findings of this study demonstrate that TMV infection significantly impairs photosynthetic efficiency and chlorophyll metabolism in tobacco, as evidenced by the marked reduction in chlorophyll content, Pn, Gs, Ci, and Tr in the CK + TMV group. However, IBC treatment effectively counteracted these adverse effects, restoring physiological parameters to levels comparable to or exceeding baseline values. Notably, the dramatic increases in Rubisco activity (115.8%) and Tr (336.8%) at 3 dpi, alongside the recovery of Pn (347.7% at 7 dpi) and chlorophyll content (100.7% at 9 dpi), suggest that IBC enhances plant resilience to viral stress by modulating key metabolic pathways.

In addition, Rubisco is also an important indicator for measuring plant photosynthesis. Rubisco directly affects the efficiency of plant photosynthesis due to its dominant role in carbon fixation and assimilation during photosynthesis [50]. Enhancing Rubisco function is essential for plant productivity and resource efficiency. Increasing Rubisco activity will improve photosynthetic efficiency and help mitigate the impact of adverse conditions on CO_2_ assimilation and crop growth. Sampol et al. [51] found that Rubisco activity was significantly reduced in virus-infected plants. The findings demonstrated that in IBC-treated tobacco leaves, Rubisco activity was significantly increased and higher than that in the CK + TMV and CK groups. This suggests that IBC can regulate plant carbon assimilation capacity by modulating Rubisco activity.

Proteomic analysis revealed that IBC mainly regulates plant photosynthesis by affecting three pathways: photosynthetic antenna proteins, photosynthesis, and photosynthesis carbon fixation. Proteomic analysis revealed that TMV-induced perturbations in protein expression were substantially mitigated by IBC. The identification of 429 DEPs between the IBC + TMV and CK + TMV groups, with 237 upregulated and 192 downregulated proteins, highlights the regulatory role of IBC in reprogramming the host proteome. The overlap of 97 DEPs between the CK + TMV vs. CK and IBC + TMV vs. CK + TMV comparisons implies a shared molecular response to TMV infection, while the distinct 324 and 332 unique DEPs reflect pathway-specific adaptations triggered by IBC.

Photosynthetic antenna proteins are crucial for capturing light energy by binding to pigment molecules to perform photosynthesis. Both PSI and PSII complexes have their own antenna proteins, whose main function is to absorb and capture light energy and transfer it to the reaction center, which is the initial step of photosynthesis. Genomic studies have shown that plants infected by TYLCV exhibit symptoms such as yellowing and curling of leaves and delayed growth and development due to significant downregulation of genes encoding LHC proteins [52]. Plant PSI complex antenna proteins mainly include five types, LHCA1, LHCA2, LHCA3, LHCA4, and LHCA5, while plant PSII complex antenna proteins mainly include seven types: LHCB1, LHCB2, LHCB3, LHCB4, LHCB5, LHCB6, and LHCB7. In the present study, there were 14 DEPs related to photosynthetic antenna proteins between the IBC + TMV and CK + TMV groups, including subunits participating in the formation of PSI antenna proteins (LHCA1, LHCA2, LHCA3, LHCA4, LHCA5) and subunits involved in the formation of PSII antenna proteins (LHCB1, LHCB2, LHCB3, LHCB4, LHCB6), and their expression levels were significantly upregulated. This suggests that IBC can regulate the expression of antenna proteins in tobacco under TMV stress, improving the plant’s ability to capture light energy.

In the light reaction of photosynthesis, the electron transport chain is mainly composed of the PSI complex, PSII complex, cytochrome b6/f complex, plastocyanin, plastoquinone, and ferredoxin. The conversion of electrical energy to chemical energy is accomplished through electron transfer, while the proton gradient generated during this process promotes the action of ATP synthase to produce ATP. Souza et al. [52] showed that viral infection reduces the expression of electron carrier proteins during photosynthesis, affecting plant photosynthesis. The data obtained in this study suggested that there were 15 DEPs between the IBC + TMV and CK + TMV groups, which constituted the subunits of PSI complex, PSII complex, cytochrome b6/f complex, ATP synthase complex, and ferredoxin, and their expression levels were significantly upregulated. The upregulation of certain protein subunits in the ETC can enhance the electron transfer rate, further increasing the generation of reducing power in plants. The enrichment of DEPs in photosynthesis-related pathways (nta00195, nta00196) and ribosome pathways (nta03010) underscores IBC’s role in preserving photosynthetic machinery. The upregulation of 14 DEPs in photosynthetic antennas, including LHCA1-5 and LHCB1-6, suggests enhanced light-harvesting capacity. These proteins are critical for energy absorption and transfer to reaction centers, which aligns with the observed recovery of Pn and Tr. Similarly, the upregulation of PSI (PsaG, PsaH, PsaK, etc.) and PSII (PsbQ, PsbE, PsbH) complex subunits implies improved electron transport efficiency, facilitating ATP/NADPH synthesis.

The dark reaction stage of photosynthesis utilizes the NADPH and ATP generated in the light reaction to perform carbon assimilation, reducing CO_2_ to sugars. Rubisco is the most critical enzyme in the dark reaction, catalyzing the fixation of CO_2_ into carboxylic acids, which are then reduced. Han et al. [53] demonstrated that viral infection reduces the levels of Rubisco large and small subunits in plants. In this study, three DEPs related to photosynthesis carbon fixation were shared between the IBC + TMV and CK + TMV groups, including Rubisco small chain clone 512-like, ribose-5-phosphate isomerase 3 (RPI3) and ribose-5-phosphate isomerase 2 (RPI2), with significantly upregulated expression levels. It is evident that IBC can promote CO_2_ fixation and ribulose-5-phosphate generation in the Calvin cycle of tobacco under TMV stress.

## 4. Materials and Methods

### 4.1. Plant Materials and Virus

Common tobacco NC89 (*Nicotiana tabacum* var. NC89), devoid of the ‘N’ resistance gene against TMV, was utilized as the host plant. Seeds of common tobacco NC89 were initially soaked in 75% ethanol for 30 to 60 s, followed by three rinses with sterile water. Subsequently, the seeds were immersed in hot water at 55 °C for 15 min and then placed in a 30 °C constant temperature water bath for 6 h once the water temperature had dropped to 30 °C. After the hot water treatment, the seeds were sown in a seedling tray filled with soil and transplanted into seedling pots when the seedlings reached the three-leaf stage. The experimental plants were cultivated under greenhouse conditions at Shenyang Tongxiang Biopesticide Co., Ltd. (Shengyang, China) (23 to 26 °C, 16/8 h light/dark cycle, 200 µmol·m^−2^·s^−1^, and humidity of 50% to 60%). The virus used in this study was a strain of TMV provided by the Plant Virus Laboratory of Shenyang Agricultural University. The virus extraction and purification were performed by inoculating NC89 with TMV, following the method described by Gooding and Hebert [54].

**Figure 7:**
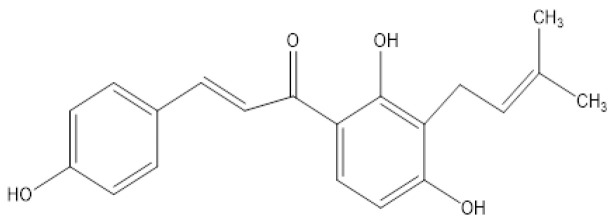
Molecular structure of IBC (Isobavachalcone).

IBC was purchased from Chengdu Pufeide Biotechnology Co., Ltd. (Chengdu, China). with a purity of ≥98%. The two-dimensional (2 D) structure is shown in Figure 7.

### 4.2. Sample Treatment

Physiological and biochemical assays were conducted using healthy NC89 tobacco plants at the 6–8 leaf stage. The experiment included three treatments: a blank control group (CK), an inoculation control group (CK + TMV), and a 40 mg/L IBC treatment group (IBC + TMV). The CK and CK + TMV groups were sprayed with an equivalent amount of water equivalent to that used in the IBC treatment group. Foliar sprays were applied three times at five-day intervals using a track-type crop sprayer operating at a pressure of 1.95 kg/cm^2^, an application rate of 50 mL/m^2^, and a track speed of 1.48 km/h. At 24 h after the third treatment, the second leaf from the middle of the tobacco plants was selected and rub-inoculated with 50 μL of TMV viral suspension at a concentration of 10 mg/L. Leaf samples were collected from the leaf immediately above the inoculated leaf at 1, 3, 5, 7, and 9 days post-inoculation (dpi). For each treatment and time point, three biological replicates were collected. All samples were rapidly frozen in liquid nitrogen and stored at -80 °C for subsequent physiological and biochemical analysis.

Whole-plant mechanical inoculation was implemented to ensure consistent viral infection across all treated plants. Following inoculation, disease severity was systematically assessed and recorded for all plants in strict accordance with the national standard “NY/T 1464.73-2018: Guidelines for Evaluation of Tobacco Mosaic Virus Disease” issued by the Ministry of Agriculture and Rural Affairs of the People’s Republic of China.

### 4.3. Determination of Chlorophyll Content

The chlorophyll content was determined according to the method of Wintermans and Mots [55] with some modifications. Fresh leaves (0.1 g) were weighed and cut into pieces in a mortar, followed by the addition of calcium carbonate, a small amount of quartz sand, and 10 mL of 95% ethanol. The mixture was ground into a homogenate and allowed to stand for 5 min before being filtered through three layers of filter paper into a 25 mL brown volumetric flask. The mortar was then rinsed with 5 mL of 95% ethanol twice. Finally, the volume was adjusted to 25 mL with 95% ethanol, and the process was repeated three times for later use. Chlorophyll a and b were quantified separately by measuring absorbance at 663 nm and 645 nm, respectively, utilizing the difference in their maximum absorption peaks in ethanol.

### 4.4. Determination of Rubisco Activity

Tobacco leaves were placed in a pre-cooled mortar and ground in an ice bath with phosphate buffer (pH 7.2, containing 0.1% polyvinylpyrrolidone) at a ratio of 1:5 (*w*/*v*) until a homogenate was obtained. The homogenate was centrifuged at 12,000× *g* for 15 min at 4 °C. The supernatant was aliquoted into centrifuge tubes and stored in a −80 °C freezer for later use.

Rubisco activity was determined according to the method of Lilley and Walker [56]. The total volume of the reaction mixture was 3 mL, containing 1.8 mL of 100 mM Tris-HCl buffer (pH 7.8, with 12 mM MgCl_2_, 0.4 mM EDTA-2Na), 0.1 mL of 160 U/mL phosphocreatine kinase solution, 0.3 mL of 50 mM ATP solution, 0.2 mL of 50 mM phosphocreatine solution, 0.1 mL of 160 U/mL phosphoglycerate kinase solution, 0.2 mL of distilled water, 0.2 mL of 0.2 M NaHCO_3_ solution, and 0.1 mL of Rubisco enzyme solution. The reaction mixture was preheated at 25 °C for 10 min, and the reaction was initiated by adding the enzyme solution. The mixture was rapidly stirred, with timing commencing immediately thereafter. The absorbance value at 340 nm was measured for 3 min, with readings taken every 30 s. One unit of enzyme activity was defined as a change in absorbance of 0.1 within 30 s.

### 4.5. Determination of Photosynthetic Parameters

The photosynthetic parameters of tobacco plants from different treatment groups were measured using an LI-6400 (LI-COR, Lincoln, NE, USA) portable photosynthesis system at 25 °C, under 1200 μmol·m^−2^·s^−1^ red–blue light sources, and in an open environment. The parameters measured included net photosynthetic rate (Pn), stomatal conductance (Gs), intercellular CO_2_ concentration (Ci), and transpiration rate (Tr) of the middle leaves of each treated plant. The leaf chamber of the photosynthesis system was equilibrated for at least 15 min under conditions of 24–26 °C to ensure a stable state before measurement. The photosynthesis measurements were taken between 9:00 and 11:00 AM on sunny days.

### 4.6. Protein Preparation

All plant samples were taken out of the frozen state and transferred to MP oscillation tubes, and an appropriate amount of phospho-protein extraction buffer (BPP) solution was added. The samples were oscillated using a high-throughput tissue grinder for 3 times, 40 s each time. The samples were centrifuged at 12,000× *g* for 20 min at 4 °C, and the supernatant was collected. An equal volume of Tris-saturated phenol was added, and the mixture was vortexed at 4 °C for 10 min. The samples were centrifuged at 12,000× *g* for 20 min at 4 °C, and the phenol phase was collected. An equal volume of BPP solution was added, and the mixture was vortexed at 4 °C for 10 min. The samples were centrifuged at 12,000× *g* for 20 min at 4 °C, and the phenol phase was collected. Five volumes of pre-cooled ammonium acetate methanol solution were added, and the proteins were precipitated overnight at −20 °C. The next day, the samples were centrifuged at 12,000× *g* for 20 min at 4 °C, and the supernatant was discarded. Pre-cooled 90% acetone was added to the precipitate, mixed, and centrifuged, and the supernatant was discarded. This step was repeated twice. Finally, the precipitate was dissolved in a protein lysis buffer (8 M urea + 1% SDS, containing protease inhibitor cocktail) to obtain a protein solution. The samples were sonicated on ice for 2 min and then centrifuged at 12,000× *g* for 20 min at 4 °C to obtain the protein supernatant. The protein concentration was determined according to the instructions provided with the Thermo Scientific (Rockford, IL, USA) Pierce BCA Protein Assay kit.

### 4.7. Reduction, Alkylation and Enzymatic Digestion

A 100 μg protein sample was taken and TEAB (Triethylammonium bicarbonate buffer, pH 8.5) was added to make the final concentration of TEAB 100 mM. TCEP (Tris(2-carboxyethyl)phosphine) was added to make the final concentration of TCEP 100 mM, and the reaction was carried out at 37 °C for 60 min. Iodoacetamide (IAM) was added to make the final concentration of IAM 40 mM, and the reaction was carried out at room temperature in the dark for 40 min. Pre-cooled acetone was added to each tube according to the volume ratio of acetone to sample of 6:1, and the samples were precipitated at −20 °C for 4 h. The samples were centrifuged at 10,000× *g* for 20 min, and the precipitate was collected. The samples were thoroughly dissolved in 100 µL of 100 mM TEAB. Trypsin was added according to the mass ratio of enzyme to protein of 1:50, and the samples were digested overnight at 37 °C.

### 4.8. TMT Labeling and Peptide Fractionation

The TMT reagent (ThermoFisher, Rockford, IL, USA) was taken out at −20 °C and brought to room temperature, centrifuged, and acetonitrile was added. The mixture was vortexed and centrifuged. One tube of TMT reagent was added for every 100 μg of peptide. The samples were incubated at room temperature for 2 h, and then hydroxylamine was added and left at room temperature for 30 min. Equal amounts of labeled products from each group were mixed into one tube and dried in a vacuum concentrator.

The peptide samples were resuspended in UPLC loading buffer (2% acetonitrile, adjusted to pH 10 with ammonia) and separated by high-pH liquid chromatography using a reversed-phase C18 column. The column specifications were 2.1 mm × 150 mm, 1.7 µm (Waters, Milford, MA, USA). Mobile phase A was 2% acetonitrile (adjusted to pH 10 with ammonia), and mobile phase B was 80% acetonitrile (adjusted to pH 10 with ammonia). Gradient elution was performed, and the UPLC elution gradient details are shown in Table 3. The gradient time was 48 min, the UV detection wavelength was 214 nm, and the flow rate was 200 μL/min. According to the time and peak shape, 28 fractions were collected and combined into 14 fractions, which were vacuum centrifuged and concentrated. The fractions were then dissolved in a mass spectrometry loading buffer (2% acetonitrile, 0.1% formic acid) for the second-dimensional analysis.

### 4.9. LC-Tandem Mass Spectrometry (MS/MS) Quantitative Proteomics Analysis

The EASY-n LC 1200 (Thermo, Sunnyvale, CA, USA) chromatography system was used for sample injection. Mobile phase A was 0.1% formic acid, and mobile phase B was 100% acetonitrile with 0.1% formic acid. The liquid phase gradient is shown in Table 4, and the flow rate was 300 nL/min. The separated components flowed out of the liquid chromatograph and entered the ion source through the interface. After ionization, they were separated in the first-stage mass spectrometry according to the mass-to-charge ratio and then analyzed by second-stage mass spectrometry. The resolution of the first stage of mass spectrometry was 60,000, the maximum injection time was 25 ms, and the fragmentation method was HCD. The second-stage resolution was 15,000, and the maximum injection time was 22 ms. Finally, the raw mass spectrometry data were obtained.

### 4.10. Database Search

The raw data were submitted to the server using Proteome Discoverer TM Software 2.4, and a pre-established database was selected for searching. During the search, the enzyme name was set to trypsin (Full), with a maximum of 2 missed cleavage sites and a precursor mass tolerance of 20 ppm. Carbamidomethyl (C), TMT pro (K), and TMT pro (N-Terminus) were set as static modifications, while Oxidation (M), Acetyl (Protein N-Terminus), Met-loss (Protein N-Terminus), and Met-loss+ Acetyl (Protein N-Terminus) were set as dynamic modifications. The validation was based on the *p*-value.

### 4.11. Bioinformatic Analysis

First, all proteins identified by mass spectrometry were compared against seven major databases (Uniprot, NR, GO, KEGG, COG, Pfam, and String) to obtain and analyze the annotation information of the proteins in each database. The proteins were also compared with databases related to subcellular localization to predict their cellular locations. In addition, GO or KEGG enrichment analysis was performed on the concentrated proteins to obtain their GO and KEGG functions. Gene Ontology (GO, http://geneontology.org/) was selected to perform GO annotation and functional clustering analysis of all differential proteins from three aspects: biological processes (BP), cellular components (CC), and molecular functions (MF); use the KEGG (Kyoto Encyclopedia of Genes and Genomes, http://www.genome.jp/kegg/ (accessed on 26 April 2022)) database to analyze the metabolic pathways involved by the differential proteins. The Goatools software (version 0.6.5) was used with Fisher’s exact test, and when the corrected *p*-value (*p*-adjust) was <0.05, the GO function or KEGG function was considered significantly enriched. When screening for differentially expressed proteins (DEPs) between groups, the screening criteria were set as FC > 1.2 for upregulated proteins, FC < 0.83 for downregulated proteins, and a *p*-value < 0.05. Proteins meeting these criteria in both samples were considered significantly DEPs.

### 4.12. Statistical Analysis

Statistical analysis was performed using SPSS 23.0. All data are expressed as the mean of three independent biological replicates. The statistical methodology employed was two-way ANOVA followed by Tukey’s multiple comparisons test. Differences were considered statistically significant at *p*-value < 0.05.

## 5. Conclusions

This study elucidates the mechanism of IBC in promoting tobacco photosynthesis under TMV stress from physiological, biochemical, and molecular levels. On the one hand, IBC treatment can increase chlorophyll content and facilitate light energy capture; on the other hand, IBC upregulates key proteins involved in photosynthetic electron transport and carbon fixation processes, thereby enhancing photosynthetic efficiency. These findings provide a theoretical basis for the application of IBC in agricultural production to control plant viral diseases and promote crop growth. In the future, further research is needed to investigate the upstream signal transduction mechanisms of IBC in regulating plant photosynthesis and its impact on plant responses to abiotic stresses in order to provide more comprehensive theoretical guidance for its widespread application in agricultural production.

## Figures and Tables

**Table 1:** Comparative analysis of TMV symptom severity between CK + TMV and IBC + TMV groups over time.

Time Point (Days)	CK + TMV	IBC + TMV
3	Vein clearing on heart leaves	No disease symptoms were observed.
5	The leaves exhibit mild mosaic symptoms, and the infected plants are stunted.	Vein clearing on heart leaves
7	Approximately 1/3 of the leaves exhibit mosaic and deformation symptoms, and the infected plants are stunted.	The heart leaves exhibited mild mosaic symptoms, and the height of the diseased plants was 1.18 times that of the CK + TMV group.
9	Approximately 1/2 to 2/3 of the leaves exhibit mosaic and deformation symptoms, and the infected plants are stunted.	Approximately 1/3 of the leaves exhibited mosaic symptoms, with a few leaves showing deformation. The height of the diseased plants was 1.27 times that of the CK + TMV group.

**Table 2:** Statistical analysis of differential genes between groups.

Different Groups	Total Protein	Up-Regulated Proteins	Down-Regulated Proteins
CK + TMV vs. CK	421	211	210
IBC + TMV vs. CK + TMV	429	237	192

**Table 3:** The gradient of UPLC.

Time (min)	B (%)
0	0
1.9	0
2	5
17	5
18	10
35.5	30
38	36
39	42
40	42
44	100
45	0
48	0

**Table 4:** The liquid phase gradient of EASY-nLC.

Time (min)	B (%)
0	5
2	5
30	38
40	90
44	Stop

## Data Availability

The original contributions presented in this study are included in the article/Supplementary Materials. Further inquiries can be directed to the corresponding author.

## Supplementary Materials

The following supporting information can be downloaded at https://www.mdpi.com/article/10.3390/plants14233638/s1. Table S1: GO enrichment analysis in IBC + TMV vs. CK + TMV; Table S2: KEGG enrichment analysis in IBC + TMV vs. CK + TMV; Table S3: Statistics of DEPs in Photosynthesis-anteinna in IBC + TMV vs. CK + TMV; Table S4: Statistics of DEPs in Photosynthesis in IBC + TMV vs. CK + TMV; Table S5: Statistics of DEPs in Carbon fixation in photosynthetic organisms in IBC + TMV vs. CK + TMV.

## Abbreviations

The following abbreviations are used in this manuscript:

IBC	isobavachalcone
TMV	tobacco mosaic virus
Pn	net photosynthetic rate
Gs	stomatal conductance
Ci	intercellular CO2 concentration
Tr	transpiration rate
dpi	days post inoculation
MYMIV	mungbean yellow mosaic India virus
AMV	alfalfa mosaic virus
LHC	light-harvesting complex
TYLCV	tomato yellow leaf curl virus
PSbMV	pea seed-borne mosaic virus
BCMV	bean common mosaic virus
DEGs	differentially expressed genes
PSII	Photosystem II
PSI	Photosystem I
ATPase	ATP synthase
MCMV	maize chlorotic mottle virus
SCMV	sugarcane mosaic virus
DEIs	differentially expressed isoforms
CYVCV	citrus yellow vein clearing virus
PVY	potato virus Y
MLT	melatonin
SA	salicylic acid
SOD	superoxide dismutase
POD	peroxidase
PPO	polyphenol oxidase
PAL	phenylalanine ammonia-lyase
CAT	catalase
BP	biological processes
CC	cellular components
MF	molecular functions
KEGG	Kyoto Encyclopedia of Genes and Genomes
DEPs	differentially expressed proteins
PC	plastocyanin
Fd	ferredoxin
FNR	ferredoxin-NADP+ reductase
cytc6	cytochrome C6
CMV	Cucumber mosaic cucumovirus
CHLG	chlorophyll synthase
CLH1	Chlorophyllase 1
CLH2	Chlorophyllase 2
PPH	pheophytin pheophorbide hydrolase
PMMoV	pepper mild mottle virus
RPI3	ribose-5-phosphate isomerase 3
RPI2	ribose-5-phosphate isomerase 2
