# Supplementary material for: Isobavachalcone Alleviates Plant Photosynthesis Inhibition Caused by Tobacco Mosaic Virus (TMV) Infection in Tobacco
Source: Plants (Basel). 2025 Nov 28;14(23):3638. doi: 10.3390/plants14233638 (PMC12694454; doi:10.3390/plants14233638)
Supplement: Supplementary file 1 [file plants-14-03638-s001.zip › Supplementary Materials.pdf]

Supplementary Materials

Title：Isobavachalcone alleviates plant photosynthesis inhibition caused by TMV infection in tobacco

Table S1: GO enrichment analysis in IBC+TMV vs CK+TMV

Number	Protein Number	GO ID	GO Description	GO Term(Level1)	Ratio_in_study	Ratio_in_pop	P_value_uncorrected	P_value_corrected
1	14	GO:0018298	protein-chromophore linkage	BP	14/408	40/5838	2.30E-07	6.59E-05
2	15	GO:0006869	lipid transport	BP	15/408	41/5838	3.27E-07	3.13E-05
3	21	GO:0009416	response to light stimulus	BP	21/408	91/5838	7.41E-07	2.66E-05
4	21	GO:0009314	response to radiation	BP	21/408	91/5838	7.41E-07	2.66E-05
5	14	GO:0009768	photosynthesis, light harvesting in photosystem I	BP	14/408	27/5838	7.97E-07	2.29E-05
6	14	GO:0009765	photosynthesis, light harvesting	BP	14/408	27/5838	7.97E-07	2.29E-05
7	69	GO:0006412	translation	BP	69/408	280/5838	1.59E-06	2.68E-05
8	72	GO:0034645	cellular macromolecule biosynthetic process	BP	72/408	391/5838	1.63E-06	2.45E-05
9	69	GO:0043043	peptide biosynthetic process	BP	69/408	282/5838	1.84E-06	2.40E-05
10	78	GO:0006518	peptide metabolic process	BP	78/408	345/5838	1.86E-06	2.32E-05
11	72	GO:0009059	macromolecule biosynthetic process	BP	72/408	418/5838	2.05E-06	2.45E-05
12	77	GO:0044271	cellular nitrogen compound biosynthetic process	BP	77/408	565/5838	2.13E-06	2.45E-05
13	69	GO:0043604	amide biosynthetic process	BP	69/408	318/5838	2.16E-06	2.38E-05
14	81	GO:1901566	organonitrogen compound biosynthetic process	BP	81/408	643/5838	2.32E-06	2.22E-05
15	99	GO:0019538	protein metabolic process	BP	99/408	787/5838	2.75E-06	2.26E-05
16	78	GO:0043603	cellular amide metabolic process	BP	78/408	415/5838	3.01E-06	2.33E-05
17	95	GO:0044267	cellular protein metabolic process	BP	95/408	610/5838	3.10E-06	2.34E-05
18	105	GO:0044260	cellular macromolecule metabolic process	BP	105/408	917/5838	3.86E-06	2.46E-05
19	8	GO:0000027	ribosomal large subunit assembly	BP	8/408	17/5838	7.38E-06	3.99E-05
20	125	GO:0043170	macromolecule metabolic process	BP	125/408	1280/5838	2.26E-05	0.000113836
21	16	GO:0006364	rRNA processing	BP	16/408	76/5838	5.30E-05	0.00023055
22	106	GO:0034641	cellular nitrogen compound metabolic process	BP	106/408	1066/5838	6.21E-05	0.000266159
23	16	GO:0016072	rRNA metabolic process	BP	16/408	80/5838	0.000101801	0.000411507
24	10	GO:0002181	cytoplasmic translation	BP	10/408	37/5838	0.000156296	0.000606175
25	5	GO:0000462	maturation of SSU-rRNA from tricistronic rRNA transcript (SSU-rRNA, 5.8S rRNA, LSU-rRNA)	BP	5/408	10/5838	0.000305567	0.001138931
26	16	GO:0015979	photosynthesis	BP	16/408	91/5838	0.000482336	0.001667838
27	28	GO:0009628	response to abiotic stimulus	BP	28/408	206/5838	0.00066341	0.002213938
28	27	GO:0006091	generation of precursor metabolites and energy	BP	27/408	205/5838	0.001164153	0.003671561
29	5	GO:0030490	maturation of SSU-rRNA	BP	5/408	13/5838	0.00130863	0.003953439
30	94	GO:0044249	cellular biosynthetic process	BP	94/408	1016/5838	0.002813092	0.007915268
31	3	GO:0015718	monocarboxylic acid transport	BP	3/408	5/5838	0.003046916	0.008489951
32	3	GO:0090391	granum assembly	BP	3/408	5/5838	0.003046916	0.008489951
33	16	GO:0034470	ncRNA processing	BP	16/408	107/5838	0.003258481	0.008822492
34	13	GO:0022618	ribonucleoprotein complex assembly	BP	13/408	80/5838	0.00332629	0.008921918
35	13	GO:0071826	ribonucleoprotein complex subunit organization	BP	13/408	81/5838	0.003717861	0.009879872

36	7	GO:0022613	ribonucleoprotein complex biogenesis	BP	7/408	30/5838	0.003849381	0.009952904
37	2	GO:0010043	response to zinc ion	BP	2/408	2/5838	0.004873049	0.011852247
38	2	GO:0072488	ammonium transmembrane transport	BP	2/408	2/5838	0.004873049	0.011852247
39	2	GO:0097753	membrane bending	BP	2/408	2/5838	0.004873049	0.011852247
40	2	GO:0015696	ammonium transport	BP	2/408	2/5838	0.004873049	0.011852247
41	29	GO:0022607	cellular component assembly	BP	29/408	248/5838	0.004961865	0.011484318
42	4	GO:0050821	protein stabilization	BP	4/408	12/5838	0.007411469	0.015413708
43	6	GO:0009767	photosynthetic electron transport chain	BP	6/408	26/5838	0.007783439	0.016070842
44	123	GO:1901564	organonitrogen compound metabolic process	BP	123/408	1434/5838	0.008590964	0.017486573
45	8	GO:0006749	glutathione metabolic process	BP	8/408	43/5838	0.008811083	0.017808316
46	94	GO:1901576	organic substance biosynthetic process	BP	94/408	1057/5838	0.009207437	0.018224375
47	4	GO:0042254	ribosome biogenesis	BP	4/408	13/5838	0.010123842	0.018506642
48	10	GO:0044085	cellular component biogenesis	BP	10/408	63/5838	0.01122589	0.019887842
49	24	GO:0006396	RNA processing	BP	24/408	205/5838	0.011342722	0.019971542
50	24	GO:0065003	protein-containing complex assembly	BP	24/408	206/5838	0.011685086	0.0204489
51	4	GO:0031647	regulation of protein stability	BP	4/408	14/5838	0.013405371	0.022499074
52	2	GO:0010597	green leaf volatile biosynthetic process	BP	2/408	3/5838	0.013941129	0.022863451
53	2	GO:0019372	lipoxygenase pathway	BP	2/408	3/5838	0.013941129	0.022863451
54	9	GO:0031324	negative regulation of cellular metabolic process	BP	9/408	56/5838	0.014605462	0.02303169
55	143	GO:0006807	nitrogen compound metabolic process	BP	143/408	1733/5838	0.015633682	0.024385145
56	4	GO:0015711	organic anion transport	BP	4/408	16/5838	0.021813537	0.031459724
57	6	GO:0065004	protein-DNA complex assembly	BP	6/408	33/5838	0.024783956	0.03403347
58	95	GO:0009058	biosynthetic process	BP	95/408	1107/5838	0.025764952	0.03504522
59	2	GO:0009269	response to desiccation	BP	2/408	4/5838	0.026596811	0.036006061
60	2	GO:0006848	pyruvate transport	BP	2/408	4/5838	0.026596811	0.036006061
61	2	GO:0006690	icosanoid metabolic process	BP	2/408	4/5838	0.026596811	0.036006061
62	2	GO:1905156	negative regulation of photosynthesis	BP	2/408	4/5838	0.026596811	0.036006061
63	2	GO:0006850	mitochondrial pyruvate transmembrane transport	BP	2/408	4/5838	0.026596811	0.036006061
64	2	GO:1901475	pyruvate transmembrane transport	BP	2/408	4/5838	0.026596811	0.036006061
65	3	GO:0046942	carboxylic acid transport	BP	3/408	10/5838	0.028097866	0.035681804
66	3	GO:1903825	organic acid transmembrane transport	BP	3/408	10/5838	0.028097866	0.035681804
67	3	GO:1905039	carboxylic acid transmembrane transport	BP	3/408	10/5838	0.028097866	0.035681804
68	57	GO:0050896	response to stimulus	BP	57/408	620/5838	0.029827492	0.036427618
69	5	GO:0006334	nucleosome assembly	BP	5/408	26/5838	0.031553822	0.038050197
70	6	GO:0071824	protein-DNA complex subunit organization	BP	6/408	35/5838	0.032311077	0.038478336
71	24	GO:0043933	protein-containing complex subunit organization	BP	24/408	225/5838	0.032594297	0.038496145
72	4	GO:0045037	protein import into chloroplast stroma	BP	4/408	18/5838	0.03284408	0.038474494
73	5	GO:0045934	negative regulation of nucleobase-containing compound metabolic process	BP	5/408	27/5838	0.036581586	0.041172216
74	3	GO:0010109	regulation of photosynthesis	BP	3/408	11/5838	0.036674962	0.04111607
75	3	GO:0009773	photosynthetic electron transport in photosystem I	BP	3/408	11/5838	0.036674962	0.04111607
76	3	GO:0031408	oxylipin biosynthetic process	BP	3/408	11/5838	0.036674962	0.04111607
77	3	GO:0031407	oxylipin metabolic process	BP	3/408	11/5838	0.036674962	0.04111607

78	5	GO:0034728	nucleosome organization	BP	5/408	28/5838	0.042074422	0.046089157
79	21	GO:0034622	cellular protein-containing complex assembly	BP	21/408	193/5838	0.042598746	0.045789663
80	4	GO:0015849	organic acid transport	BP	4/408	20/5838	0.046600256	0.048990013
81	9	GO:0022900	electron transport chain	BP	9/408	66/5838	0.046868546	0.049092236
82	5	GO:0016567	protein ubiquitination	BP	5/408	29/5838	0.048038229	0.049063957
83	15	GO:0019219	regulation of nucleobase-containing compound metabolic process	BP	15/408	127/5838	0.049552483	0.049725743
84	9	GO:0034220	ion transmembrane transport	BP	9/408	67/5838	0.049662532	0.049662532
85	18	GO:0022627	cytosolic small ribosomal subunit	CC	18/408	53/5838	3.25E-07	4.66E-05
86	37	GO:0015934	large ribosomal subunit	CC	37/408	135/5838	4.36E-07	3.13E-05
87	22	GO:0009521	photosystem	CC	22/408	48/5838	4.62E-07	2.65E-05
88	19	GO:0009522	photosystem I	CC	19/408	37/5838	5.95E-07	2.85E-05
89	33	GO:0022625	cytosolic large ribosomal subunit	CC	33/408	113/5838	6.14E-07	2.52E-05
90	17	GO:0009523	photosystem II	CC	17/408	38/5838	8.48E-07	2.03E-05
91	21	GO:0015935	small ribosomal subunit	CC	21/408	78/5838	1.04E-06	2.30E-05
92	68	GO:1990904	ribonucleoprotein complex	CC	68/408	336/5838	1.28E-06	2.45E-05
93	58	GO:0044391	ribosomal subunit	CC	58/408	213/5838	1.42E-06	2.55E-05
94	60	GO:0005840	ribosome	CC	60/408	241/5838	1.62E-06	2.59E-05
95	72	GO:0043228	non-membrane-bounded organelle	CC	72/408	369/5838	2.29E-06	2.35E-05
96	72	GO:0043232	intracellular non-membrane-bounded organelle	CC	72/408	369/5838	2.29E-06	2.35E-05
97	133	GO:0032991	protein-containing complex	CC	133/408	1104/5838	2.82E-06	2.25E-05
98	44	GO:0042170	plastid membrane	CC	44/408	314/5838	6.09E-06	3.43E-05
99	48	GO:0098796	membrane protein complex	CC	48/408	354/5838	7.11E-06	3.92E-05
100	4	GO:0098807	chloroplast thylakoid membrane protein complex	CC	4/408	4/5838	2.35E-05	0.00011446
101	4	GO:0030093	chloroplast photosystem I	CC	4/408	4/5838	2.35E-05	0.00011446
102	35	GO:0009535	chloroplast thylakoid membrane	CC	35/408	241/5838	2.55E-05	0.000119987
103	35	GO:0055035	plastid thylakoid membrane	CC	35/408	241/5838	2.55E-05	0.000119987
104	35	GO:0042651	thylakoid membrane	CC	35/408	244/5838	4.80E-05	0.000215418
105	35	GO:0034357	photosynthetic membrane	CC	35/408	244/5838	4.80E-05	0.000215418
106	361	GO:0005575	cellular_component	CC	361/408	4757/5838	9.12E-05	0.000384735
107	3	GO:0009515	granal stacked thylakoid	CC	3/408	3/5838	0.000339009	0.001186532
108	344	GO:0110165	cellular anatomical entity	CC	344/408	4556/5838	0.00123507	0.003852882
109	65	GO:0031090	organelle membrane	CC	65/408	669/5838	0.004679917	0.011479797
110	7	GO:0010287	plastoglobule	CC	7/408	33/5838	0.006736939	0.014322234
111	3	GO:0000785	chromatin	CC	3/408	7/5838	0.009592198	0.01823153
112	7	GO:0044815	DNA packaging complex	CC	7/408	36/5838	0.010986625	0.019707259
113	7	GO:0000786	nucleosome	CC	7/408	36/5838	0.010986625	0.019707259
114	7	GO:0032993	protein-DNA complex	CC	7/408	37/5838	0.01275962	0.021797684
115	4	GO:0030684	preribosome	CC	4/408	14/5838	0.013405371	0.022499074
116	4	GO:0033177	proton-transporting two-sector ATPase complex, proton-transporting domain	CC	4/408	16/5838	0.021813537	0.031459724
117	2	GO:0045283	fumarate reductase complex	CC	2/408	4/5838	0.026596811	0.036006061
118	2	GO:0045281	succinate dehydrogenase complex	CC	2/408	4/5838	0.026596811	0.036006061
119	2	GO:0005749	mitochondrial respiratory chain complex II, succinate dehydrogenase complex (ubiquinone)	CC	2/408	4/5838	0.026596811	0.036006061

120	2	GO:0045257	succinate dehydrogenase complex (ubiquinone)	CC	2/408	4/5838	0.026596811	0.036006061
121	3	GO:0009538	photosystem I reaction center	CC	3/408	10/5838	0.028097866	0.035681804
122	3	GO:0048471	perinuclear region of cytoplasm	CC	3/408	10/5838	0.028097866	0.035681804
123	3	GO:0032040	small-subunit processome	CC	3/408	10/5838	0.028097866	0.035681804
124	7	GO:0098803	respiratory chain complex	CC	7/408	45/5838	0.034873998	0.039560623
125	10	GO:0098800	inner mitochondrial membrane protein complex	CC	10/408	76/5838	0.041472396	0.045603746
126	2	GO:0000220	vacuolar proton-transporting V-type ATPase, V0 domain	CC	2/408	5/5838	0.042296742	0.04615652
127	2	GO:0022626	cytosolic ribosome	CC	2/408	5/5838	0.042296742	0.04615652
128	115	GO:0016021	integral component of membrane	CC	115/408	1404/5838	0.047338011	0.048870537
129	14	GO:0016168	chlorophyll binding	MF	14/408	33/5838	1.07E-06	2.18E-05
130	73	GO:0005198	structural molecule activity	MF	73/408	298/5838	1.72E-06	2.48E-05
131	71	GO:0003735	structural constituent of ribosome	MF	71/408	270/5838	1.79E-06	2.45E-05
132	6	GO:0046943	carboxylic acid transmembrane transporter activity	MF	6/408	15/5838	0.000326585	0.001186453
133	6	GO:0005342	organic acid transmembrane transporter activity	MF	6/408	15/5838	0.000326585	0.001186453
134	4	GO:0031625	ubiquitin protein ligase binding	MF	4/408	7/5838	0.000694409	0.002290751
135	3	GO:0031386	protein tag	MF	3/408	4/5838	0.001285446	0.003966915
136	3	GO:0008028	monocarboxylic acid transmembrane transporter activity	MF	3/408	4/5838	0.001285446	0.003966915
137	4	GO:0044389	ubiquitin-like protein ligase binding	MF	4/408	8/5838	0.001312417	0.003923579
138	3	GO:0004869	cysteine-type endopeptidase inhibitor activity	MF	3/408	5/5838	0.003046916	0.008489951
139	12	GO:0019843	rRNA binding	MF	12/408	72/5838	0.00381296	0.009948358
140	76	GO:0003676	nucleic acid binding	MF	76/408	807/5838	0.00465687	0.011621927
141	7	GO:0003682	chromatin binding	MF	7/408	31/5838	0.004679852	0.0115786
142	2	GO:0050833	pyruvate transmembrane transporter activity	MF	2/408	2/5838	0.004873049	0.011852247
143	2	GO:0008519	ammonium transmembrane transporter activity	MF	2/408	2/5838	0.004873049	0.011852247
144	5	GO:0031490	chromatin DNA binding	MF	5/408	17/5838	0.004982972	0.011440903
145	7	GO:0004364	glutathione transferase activity	MF	7/408	32/5838	0.005638526	0.01274218
146	20	GO:0046906	tetrapyrrole binding	MF	20/408	153/5838	0.005678757	0.012732838
147	14	GO:0008289	lipid binding	MF	14/408	94/5838	0.006359796	0.013827739
148	3	GO:0004784	superoxide dismutase activity	MF	3/408	7/5838	0.009592198	0.01823153
149	3	GO:0043024	ribosomal small subunit binding	MF	3/408	7/5838	0.009592198	0.01823153
150	3	GO:0016721	oxidoreductase activity, acting on superoxide radicals as acceptor	MF	3/408	7/5838	0.009592198	0.01823153
151	5	GO:0030414	peptidase inhibitor activity	MF	5/408	20/5838	0.010493531	0.019061034
152	5	GO:0004866	endopeptidase inhibitor activity	MF	5/408	20/5838	0.010493531	0.019061034
153	5	GO:0061135	endopeptidase regulator activity	MF	5/408	21/5838	0.01300077	0.022078231
154	5	GO:0008514	organic anion transmembrane transporter activity	MF	5/408	21/5838	0.01300077	0.022078231
155	2	GO:0042879	aldonate transmembrane transporter activity	MF	2/408	3/5838	0.013941129	0.022863451
156	2	GO:0004512	inositol-3-phosphate synthase activity	MF	2/408	3/5838	0.013941129	0.022863451
157	2	GO:0045158	electron transporter, transferring electrons within cytochrome b6/f complex of photosystem II activity	MF	2/408	3/5838	0.013941129	0.022863451
158	3	GO:0015171	amino acid transmembrane transporter activity	MF	3/408	8/5838	0.014560067	0.023086958
159	58	GO:0003723	RNA binding	MF	58/408	612/5838	0.014765476	0.023156784
160	5	GO:0061134	peptidase regulator activity	MF	5/408	22/5838	0.015883376	0.024640697
161	3	GO:0019904	protein domain specific binding	MF	3/408	9/5838	0.020723747	0.030817178

162	3	GO:0004602	glutathione peroxidase activity	MF	3/408	9/5838	0.020723747	0.030817178
163	2	GO:0016630	protochlorophyllide reductase activity	MF	2/408	4/5838	0.026596811	0.036006061
164	2	GO:0004751	ribose-5-phosphate isomerase activity	MF	2/408	4/5838	0.026596811	0.036006061
165	35	GO:0022857	transmembrane transporter activity	MF	35/408	350/5838	0.029806885	0.036558018
166	4	GO:0031491	nucleosome binding	MF	4/408	18/5838	0.03284408	0.038474494
167	6	GO:0003690	double-stranded DNA binding	MF	6/408	36/5838	0.036575967	0.041327963
168	3	GO:0016682	oxidoreductase activity, acting on diphenols and related substances as donors, oxygen as acceptor	MF	3/408	11/5838	0.036674962	0.04111607
169	2	GO:0003713	transcription coactivator activity	MF	2/408	5/5838	0.042296742	0.04615652
170	2	GO:0043495	protein-membrane adaptor activity	MF	2/408	5/5838	0.042296742	0.04615652
171	19	GO:0003677	DNA binding	MF	19/408	175/5838	0.049325055	0.049671196

Table S2: KEGG enrichment analysis in IBC+TMV vs CK+TMV

Number	Protein Number	Pathway_ID	KEGG Description	Ratio_in_study	Ratio_in_pop	P_value_uncorrected	P_value_corrected
1	68	nta03010	Ribosome	68/278	253/4171	6.66E-26	4.66E-24
2	14	nta00196	Photosynthesis - antenna proteins	14/278	27/4171	2.27E-10	7.94E-09
3	15	nta00195	Photosynthesis	15/278	86/4171	0.000455257	0.010622663
4	11	nta00480	Glutathione metabolism	11/278	83/4171	0.020670757	0.361738256
5	13	nta00190	Oxidative phosphorylation	13/278	115/4171	0.040812888	0.571380432
6	6	nta00350	Tyrosine metabolism	6/278	45/4171	0.07575681	0.757568103
7	6	nta04145	Phagosome	6/278	52/4171	0.129583258	0.824620734
8	2	nta00591	Linoleic acid metabolism	2/278	7/4171	0.074384494	0.8678191
9	3	nta00590	Arachidonic acid metabolism	3/278	19/4171	0.128863297	0.90204308
10	7	nta04626	Plant-pathogen interaction	7/278	62/4171	0.116077317	0.90282358
11	4	nta04075	Plant hormone signal transduction	4/278	28/4171	0.112192015	0.981680134
12	2	nta01240	Biosynthesis of cofactors	2/278	234/4171	0.99999887	0.99999887
13	5	nta00592	alpha-Linolenic acid metabolism	5/278	45/4171	0.177664956	1
14	3	nta00941	Flavonoid biosynthesis	3/278	24/4171	0.212427142	1
15	3	nta03008	Ribosome biogenesis in eukaryotes	3/278	24/4171	0.212427142	1
16	3	nta00950	Isoquinoline alkaloid biosynthesis	3/278	25/4171	0.230377599	1
17	2	nta00945	Stilbenoid, diarylheptanoid and gingerol biosynthesis	2/278	14/4171	0.238549067	1
18	4	nta04016	MAPK signaling pathway - plant	4/278	38/4171	0.245273468	1
19	1	nta00430	Taurine and hypotaurine metabolism	1/278	5/4171	0.291815104	1
20	1	nta00909	Sesquiterpenoid and triterpenoid biosynthesis	1/278	6/4171	0.339072762	1
21	1	nta03410	Base excision repair	1/278	7/4171	0.383187473	1
22	10	nta04141	Protein processing in endoplasmic reticulum	10/278	139/4171	0.44900509	1
23	4	nta00860	Porphyrin and chlorophyll metabolism	4/278	52/4171	0.460003411	1
24	1	nta00261	Monobactam biosynthesis	1/278	9/4171	0.462807584	1
25	3	nta04120	Ubiquitin mediated proteolysis	3/278	38/4171	0.469786571	1
26	2	nta00513	Various types of N-glycan biosynthesis	2/278	24/4171	0.482139257	1
27	2	nta00510	N-Glycan biosynthesis	2/278	25/4171	0.504031575	1
28	1	nta00300	Lysine biosynthesis	1/278	11/4171	0.532182253	1
29	1	nta00073	Cutin, suberine and wax biosynthesis	1/278	13/4171	0.592625711	1
30	3	nta00240	Pyrimidine metabolism	3/278	46/4171	0.600741862	1
31	2	nta00562	Inositol phosphate metabolism	2/278	30/4171	0.60414037	1
32	4	nta04146	Peroxisome	4/278	63/4171	0.613740946	1
33	2	nta03060	Protein export	2/278	35/4171	0.68824695	1
34	1	nta00760	Nicotinate and nicotinamide metabolism	1/278	18/4171	0.711825913	1
35	2	nta00900	Terpenoid backbone biosynthesis	2/278	37/4171	0.717547312	1
36	3	nta03013	Nucleocytoplasmic transport	3/278	55/4171	0.720451454	1
37	1	nta03030	DNA replication	1/278	19/4171	0.73111616	1
38	1	nta00100	Steroid biosynthesis	1/278	19/4171	0.73111616	1
39	2	nta00220	Arginine biosynthesis	2/278	38/4171	0.731311798	1

40	2	nta00360	Phenylalanine metabolism	2/278	42/4171	0.780800267	1
41	3	nta03015	mRNA surveillance pathway	3/278	61/4171	0.783512264	1
42	3	nta03018	RNA degradation	3/278	64/4171	0.810377985	1
43	3	nta00250	Alanine, aspartate and glutamate metabolism	3/278	64/4171	0.810377985	1
44	1	nta00960	Tropane, piperidine and pyridine alkaloid biosynthesis	1/278	26/4171	0.834528652	1
45	1	nta00920	Sulfur metabolism	1/278	28/4171	0.85598274	1
46	2	nta00410	beta-Alanine metabolism	2/278	50/4171	0.856353469	1
47	1	nta00910	Nitrogen metabolism	1/278	29/4171	0.865646461	1
48	3	nta00260	Glycine, serine and threonine metabolism	3/278	76/4171	0.89134208	1
49	1	nta00460	Cyanoamino acid metabolism	1/278	33/4171	0.898255272	1
50	5	nta03040	Spliceosome	5/278	118/4171	0.903613382	1
51	3	nta00230	Purine metabolism	3/278	79/4171	0.906021632	1
52	4	nta00940	Phenylpropanoid biosynthesis	4/278	100/4171	0.909466826	1
53	1	nta00650	Butanoate metabolism	1/278	35/4171	0.911468466	1
54	4	nta00710	Carbon fixation in photosynthetic organisms	4/278	105/4171	0.927671548	1
55	1	nta00040	Pentose and glucuronate interconversions	1/278	38/4171	0.928151061	1
56	1	nta00130	Ubiquinone and other terpenoid-quinone biosynthesis	1/278	38/4171	0.928151061	1
57	1	nta00400	Phenylalanine, tyrosine and tryptophan biosynthesis	1/278	40/4171	0.937492701	1
58	2	nta00030	Pentose phosphate pathway	2/278	71/4171	0.955920859	1
59	1	nta00564	Glycerophospholipid metabolism	1/278	46/4171	0.958858537	1
60	3	nta00630	Glyoxylate and dicarboxylate metabolism	3/278	107/4171	0.977809478	1
61	1	nta00053	Ascorbate and aldarate metabolism	1/278	57/4171	0.980921637	1
62	1	nta01210	2-Oxocarboxylic acid metabolism	1/278	57/4171	0.980921637	1
63	8	nta01230	Biosynthesis of amino acids	8/278	228/4171	0.988230137	1
64	2	nta00500	Starch and sucrose metabolism	2/278	97/4171	0.99071687	1
65	1	nta04144	Endocytosis	1/278	90/4171	0.998121757	1
66	82	nta01100	Metabolic pathways	82/278	1582/4171	0.999063563	1
67	1	nta00270	Cysteine and methionine metabolism	1/278	104/4171	0.999301641	1
68	2	nta00010	Glycolysis / Gluconeogenesis	2/278	147/4171	0.999610508	1
69	5	nta01200	Carbon metabolism	5/278	309/4171	0.999996311	1
70	33	nta01110	Biosynthesis of secondary metabolites	33/278	928/4171	0.999998508	1

Table S3: Statistics of DEPs in Photosynthesis-anteinna in IBC+TMV vs CK+TMV

Accession	KO name	Description	expression quantity			IBC/CK+TMV		CK+TMV/CK	
			CK	TMV	IBC	FC	significance	FC	significance
XP_016513630.1	LHCA4LHCA2	chlorophyll a-b binding protein 4	1.013	0.862	1.54	1.787	yes	0.851	no
XP_016472945.1	LHCB2	chlorophyll a-b binding protein 36	0.95	0.775	1.379	1.779	yes	0.816	yes
XP_016469705.1	LHCB6LHCA2	chlorophyll a-b binding protein CP24 10A	0.67	1.014	1.715	1.691	yes	1.513	no
XP_016490031.1	LHCB2	chlorophyll a-b binding protein 36	0.973	0.922	1.554	1.685	yes	0.948	no
XP_016513581.1	LHCB6LHCA2	chlorophyll a-b binding protein CP24 10A	0.991	1.056	1.625	1.539	yes	1.066	no
XP_016487829.1	LHCB1	chlorophyll a-b binding protein 21	1.09	1.36	1.959	1.44	yes	1.248	yes
XP_016457506.1	LHCA2LHCA3	chlorophyll a-b binding protein 8	1.009	1.027	1.442	1.404	yes	1.018	no
XP_016490560.1	LHCB4LHCA1	chlorophyll a-b binding protein 6A	0.997	0.917	1.251	1.365	yes	0.92	no
XP_016490806.1	LHCB1	chlorophyll a-b binding protein 21	1.046	1.246	1.59	1.276	yes	1.192	no
XP_016487830.1	LHCB1	chlorophyll a-b binding protein 21	1.036	1.157	1.466	1.267	yes	1.117	no
XP_016491826.1	LHCA4LHCA2	chlorophyll a-b binding protein P4	1.078	1.035	1.31	1.266	yes	0.96	no
XP_016501042.1	LHCA2	chlorophyll a-b binding protein	1.032	1.054	1.309	1.242	yes	1.021	no
XP_016450586.1	LHCA5 LHCA4	chlorophyll a-b binding protein 5	0.994	0.908	1.123	1.236	yes	0.913	no
XP_016467476.1	LHCB2 LHCB3	chlorophyll a-b binding protein 13	1.013	1.046	1.259	1.204	yes	1.032	no

Table S4: Statistics of DEPs in Photosynthesis in IBC+TMV vs CK+TMV

Accession	KO name	Description	expression quantity			IBC/CK+TMV		CK+TMV/CK	
			CK	TMV	IBC	FC	significance	FC	significance
XP_016443581.1	PsaL	photosystem I reaction center subunit XI	0.994	1.012	1.561	1.542	yes	1.018	no
XP_016441914.1	PsaO	photosystem I subunit	0.97	0.878	1.333	1.519	yes	0.905	no
XP_016443582.1	PsaL	photosystem I reaction center subunit XI	1.027	1.101	1.628	1.479	yes	1.072	no
XP_016456381.1	PetF	ferredoxin	0.97	0.86	1.264	1.469	yes	0.887	no
XP_016509017.1	PsaG	photosystem I reaction center subunit V	0.983	0.88	1.291	1.467	yes	0.896	no
XP_016481186.1	atpF1	ATP synthase subunit b', chloroplastic-like	0.976	0.918	1.296	1.412	yes	0.941	no
XP_016443569.1	PsaK	photosystem I reaction center subunit	1.02	0.793	1.078	1.359	yes	0.778	no
XP_016442524.1	PsbQ	oxygen-evolving enhancer protein 3-2	1.005	0.823	1.094	1.33	yes	0.819	no
NP_054517.1	PsbE	photosystem II protein V	1.029	0.948	1.247	1.316	yes	0.92	no
XP_016433223.1	PsaN	photosystem I reaction center subunit N	1.011	1.077	1.401	1.301	yes	1.065	no
XP_016458078.1	atpF2	ATP synthase subunit b', chloroplastic-like	0.992	0.965	1.222	1.266	yes	0.973	no
XP_016479165.1	PsaH	photosystem I reaction center subunit VI-1	0.99	0.922	1.167	1.266	yes	0.931	no
NP_054530.1	CYTB petB (plastid)	cytochrome b6	1.052	1.025	1.284	1.253	yes	0.974	no
XP_016443822.1	CYTB PetB PsbH	cytochrome b6	0.979	0.871	1.082	1.242	yes	0.89	no
XP_016443682.1	petF	ferredoxin	0.929	1.129	1.361	1.205	yes	1.215	yes

Table S5: Statistics of DEPs in Carbon fixation in photosynthetic organisms in IBC+TMV vs CK+TMV

Accession	KO name	Description	expression quantity			IBC/CK+TMV		CK+TMV/CK	
			CK	TMV	IBC	FC	significance	FC	significance
XP_016451271.1	rpiA	ribose-5-phosphate isomerase 3	0.978	0.914	1.395	1.526	yes	0.935	no
XP_016450904.1	rbcS, cbbS	carboxylase small chain clone 512-like isoform X1	0.988	1.17	1.595	1.363	yes	1.184	no
XP_016463832.1	rpiA	ribose-5-phosphate isomerase 2	1.009	1.041	1.293	1.242	yes	1.032	no
